# Is the β Common Receptor the Key Molecule for the Protective Effect of Erythropoietin?

**DOI:** 10.14336/AD.2022.0823

**Published:** 2023-04-01

**Authors:** Zhenhong Yang, Rongliang Wang, Yangmin Zheng, Yumin Luo

**Affiliations:** ^1^Institute of Cerebrovascular Disease Research and Department of Neurology, Xuanwu Hospital of Capital Medical University, Beijing, China.; ^2^Beijing Geriatric Medical Research Center and Beijing Key Laboratory of Translational Medicine for Cerebrovascular Diseases, Beijing, China.; ^3^Beijing Institute for Brain Disorders, Capital Medical University, Beijing, China

**Keywords:** erythropoietin, β common receptor;, ischemic stroke, myocardial infarction, EPO heteroreceptor

## Abstract

Erythropoietin is generally assumed to have protective effects against multiple diseases, especially ischemic stroke, and myocardial infarctions. The theory behind Erythropoietin’s (EPO) protective effects has been misconstrued in the scientific community to a degree, with assumptions made that the β common receptor (βcR) in the heteroreceptor EPO receptor (EPOR)/βcR is responsible for these protective effects. Our purpose with this opinion article is to convey our concern for the general assumption of the importance of βcR in EPO’s protective effect and to emphasize the necessity of further research in this field.

Erythropoietin is theorized to be protective against multiple diseases, especially ischemic stroke and cardiovascular disease [1, 2]. However, the erythropoietic mechanism is a high-risk factor for patients who suffer from cardiovascular or cerebrovascular disease. It was previously discovered that EPO exerts its protective effect by binding with the EPOR/βcR heteroreceptor, while (EPOR)_2_ is responsible for hematopoietic function [3]. This discovery has inspired many follow-up studies.

## EPOR/βcR heteroreceptor and activator

In 2008, Michael Brines et al. claimed to have identified the tissue-protective domain, helix B (amino acid residues 58-82), within the EPO molecule. Helix B does not facilitate EPO/(EPOR)_2_ binding. The research team modified helix B and named the final product pyroglutamate helix B surface peptide (pHBSP). They verified its protective effects in models of ischemic stroke and renal ischemia-reperfusion and made sure pHBSP possesses no erythropoietic effect [4].

In view of many experiments of medication targeting the EPOR/βcR heteroreceptor, it is increasingly accepted that EPOR/βcR mediates the protective function of EPO. Alternative names such as tissue-protective receptor and innate immune receptor were proposed to name EPOR/βcR heteroreceptor [5, 6].

## The βcR role in the protective effect of EPO

The importance of the EPOR/βcR heteroreceptor in the protective effects of EPO is generously overestimated. According to the presumption from Michael Brines’ team, helix B of EPO faces the opposite side from the binding domain between EPO and (EPOR)_2_. Therefore, they reasoned that helix B is the key fragment for EPO’s protective effect and declared that pHBSP achieved its protective function *via* activating EPOR/βcR heteroreceptorr [4]. However, other EPO fragments utilized in EPO/(EPOR)_2_ binding sites are also shown to be protective in multiple diseases.

Two binding sites are responsible for EPO/(EPOR)_2_ binding. The EPO fragment, EPObis, highly overlaps with the site 1 binding points and consists of No. 36-53 amino acids of EPO. This fragment was found to promote the neurite outgrowth of primary neurons [7]. The fragment EPOtris is composed of No. 92-111 amino acids of EPO, with 9 binding points at site 2 included in the EPOtris sequence. EPOtris decreased the mortality of mice with brain injury and ameliorated seizure symptoms and neurodegeneration [8]. Both EPOtris and EPObis bind directly to (EPOR)_2_. Besides the previously mentioned two fragments, other fragments, namely MK-X and ML1-h3, which are derived from helix C of EPO, also bind with (EPOR)_2_ to protect brain against ischemic injury [9, 10].

These examples demonstrate that several different fragments within the EPO molecule possess protective potency. βcR is not essential in these situations.

## The fragility of the EPOR/βcR heteroreceptor hypothesis

### 1.βcR and the function of EPO, Interleukin (IL)-3, and granulocyte-macrophage colony-stimulating factor (GM-CSF)

βcR is a receptor subunit for IL-3/IL-5/GM-CSF. βcR knockout eliminates the effects of EPO and these three cytokines. Moreover, IL-3 and GM-CSF play important roles in many diseases that EPO influences.

As early as 1990, IL-3 was found to facilitate neurite outgrowth and activate choline acetyltransferase *in vitro* [11]. TC Wen et al. found that IL-3 helped attenuate ischemia-induced neuronal death, especially in the CA1 region [12].

Furthermore, Wolf-Rüdiger Schäbitz et al. verified the protective role of GM-CSF in the ischemic brain of two types of ischemic stroke models [13]. In 2019, Xianmei Li et al. found that GM-CSF significantly decreased in the serum of severe stroke patients [14].

Considering the complicated role of βcR, it is illogical to infer the direct interaction between EPO and βcR if the effects of IL-3 and GM-CSF are eliminated. Merely conducting βcR knockout is not enough to investigate the intricate relationship.

### 2. Beneficial outcomes of βcR knockout

Considering the protective role that βcR plays, theoretically, the knockout of βcR should thus prove to be detrimental. However, two articles reported that βcR knockout resulted in beneficial outcomes [15, 16].

βcR-deficient mice were more resistant to myocardial infarction. With the strong MI-resistant effects elicited by βcR knockout, routine MI surgical interventions barely influenced the cardiac hemo-dynamics of βcR-deficient mice, and post-surgical administration of darbepoetin (an erythropoietin derivative) failed to improve the already normal cardiac function of these mice further. Impaired cardiac hemodynamics was finally achieved in βcR-deficient mice by permanent coronary ligation. Moreover, darbepoetin was found to be protective against MI without the existence of the βcR. Thus, the author claimed that the βcR did not influence the positive effects of darbepoetin [16]. In 2017, the same phenomenon appeared in another experiment: the authors demonstrated that myocardial ischemia provoked fibroblastic secretion of GM-CSF, and sequentially inflammatory and proteolytic cells accumulated. Abolishing either GM-CSF or βcR resulted in fewer accumulating inflammatory cells and better heart function [15].

Therefore, we can deduce that the impedance of detrimental endogenous GM-CSF could account for the avoided injury that occurred after βcR knockout. We can therefore infer through these two experiments that βcR mediates detrimental effects.

## Conclusion

In conclusion, there are three noteworthy reasons for doubting the importance of the βcR in EPO’s protective effect. Firstly, the βcR only provides protective effects when bound to the helix B peptide and not to any other EPO fragments. Secondly, βcR knockout does not shed light on the relationship between the EPOR/βcR heteroreceptor and EPO, Thirdly, animals conversely gained resistance against injury after βcR knockout.
